# Relevance of histamine and tryptase concentrations in nasal secretions after nasal challenges with phosphate buffered saline and allergen

**DOI:** 10.1155/S0962935195000160

**Published:** 1995-03

**Authors:** D. Wang, P. Clement, J. Smitz, M.-P. Derde

**Affiliations:** 1 Department of Otorhinolaryngology University Hospital Free University Brussels Brussels Belgium; 2 Department of Radioimmunology University Hospital Free University Brussels Brussels Belgium; 3 Department of Biomedical Statistics University Hospital Free University Brussels Brussels Belgium

## Abstract

In this prospective study, a quantitative determination of histamine and tryptase in nasal secretions after nasal phosphate buffered saline (PBS) and allergen challenge was performed in 18 atopic patients who were compared with ten non-allergic healthy volunteers. The aim of the study was to determine the normal and pathological concentrations of these important mediators in nasal secretions. The second objective was to test the relevance of these two mast cell secreted mediators after nasal challenge. Results showed that the concentrations of tryptase in almost all samples were under the minimal detection limit (< 0.5 μU/g) and only a sigrtificant increase of tryptase (median, 28 μU/g) occurred immediately after nasal allergen challenge in the patient group. Histamine concentration significantly increased after every nasal PBS challenge (median, 69 ng/g after first PBS challenge and 165 ng/g after second PBS challenge) in the control group, as well as in the patient group after both PBS (median, 69 ng/g) and allergen (median, 214 ng/g) challenge. On the other hand, a rapid onset of sneezing and increase in nasal airway resistance was experienced only in the patient group after nasal allergen challenge, but did not occur after PBS challenge even though the histamine concentrations significantly increased in both groups. This study suggests that tryptase is a more preferable marker than histamine in quantitative monitoring of mast cell activation especially during the early phase nasal allergic reaction.


Mediators of Inflammation 4, 98–102 (1995)

### Relevance of histamine and tryptase concentrations in nasal secretions after nasal challenges with phosphate buffered saline and allergen

D. Wang,[1,CA] P. Clement,[1] J. Smitz[2] and M.-P. Derde[3]

[1]Department of Otorhinolaryngology, [2]Department of Radioimmunology, and [3]Department of Biomedical Statistics, University Hospital, Free University Brussels, Brussels, Belgium

[CA] Corresponding Author

**In this prospective study, a quantitative determination of histamine and tryptase in nasal secretions after nasal phosphate buffered saline (PBS) and allergen challenge was performed in 18 atopic patients who were compared with ten non-allergic healthy volunteers. The aim of the study was to determine the normal and pathological concentrations of these important mediators in nasal secretions. The second objective was to test the relevance of these two mast cell secreted mediators after nasal challenge. Results showed that the concentrations of tryptase in almost all samples were under the minimal detection limit (< 0.5 μU/g) and only a significant increase of tryptase (median, 28 μU/g) occurred immediately after nasal allergen challenge in the patient group. Histamine concentration significantly increased after every nasal PBS challenge (median, 69 ng/g after first PBS challenge and 165 ng/g after second PBS challenge) in the control group, as well as in the patient group after both PBS (median, 69 ng/g) and allergen (median, 214 ng/g) challenge. On the other hand, a rapid onset of sneezing and increase in nasal airway resistance was experienced only in the patient group after nasal allergen challenge, but did not occur after PBS challenge even though the histamine concentrations significantly increased in both groups. This study suggests that tryptase is a more preferable marker than histamine in quantitative monitoring of mast cell activation especially during the early phase nasal allergic reaction.**

**Key words**: Allergic rhinitis, Concentration, Early phase, Histamine, Nasal challenge, Nasal secretions, Tryptase

### Introduction

It has been a well-established fact for many years that histamine and tryptase are both mast cell products. Increases of these two components in body fluids have been regarded as markers for mast cell activation in allergic reactions.[1–3] Numerous clinical and experimental models have demonstrated that histamine is a most important mediator during the early-phase nasal allergic reaction.

Until now, measurable histamine levels has been found not only in plasma and urine but also in tears, gastric juice, blister fluid and cerebral spinal fluid. While the published data give the normal and pathological concentrations of histamine in body fluids,[4] the quantification of histamine in nasal secretions, however, has not yet been well established. The most common technique in use until now has been nasal lavage. The drawback of this technique, however, is that the dilutional effect of nasal secretions by lavage fluid is unknown.[5] Therefore, the important question regarding the exact normal and pathological concentrations of histamine in nasal secretions still remains unanswered.

In this study the authors used the nasal microsuction technique as a sampling tool for determination of histamine and tryptase levels in nasal secretions after nasal allergen challenge (NAC). The aim of the study was to obtain quantitative data of these two components in nasal secretions during allergen-induced nasal allergic reaction compared with the baseline values. The second objective was to test the relevance of these two markers of mast cell activation after nasal challenge, with routine negative control solution of PBS and allergen.

### Materials and Methods

*Patients:* Eighteen patients (males, eleven; females, seven) aged from 18 to 45 years (mean age, 28 years) had nasal atopy to grass pollen confirmed by medical history, skin test and nasal grass pollen challenge. Eleven of the 18 patients also had atopy to house dust and/or mites as evidenced by skin test. However, they were all symptom-free and they had a normal nasal airway resistance (NAR) as confirmed by passive anterior rhinomanometry (PAR). A control

© 1995 Rapid Communications of Oxford Ltd group consisted of ten healthy volunteers (males, five; females, five) aged from 20 to 43 years (mean age, 28 years). All of them (in both groups) had normal findings on rhinoscopy, routine blood chemistry parameters and haematological examinations. They had no disease of the respiratory tract, nor had they taken any medication (except oral contraceptives) during the previous 3 months. The study was performed 3 months after the pollen season. The subjects gave their informed consent. This study was approved by the local ethics committee.

*The aspiration system:* The authors used the Biewenga's aspiration system as described previously.[6] The samples were collected by repeated aspiration from the middle meatus and from the floor of both nasal cavities, into a pre-weighed plastic sampling tube. This was immediately followed by aspiration of a known volume (1.0 ml) of PBS (pH 7.4) including 10% of Mesna (Mistabron, UCB Pharmaceutics Company, Belgium). Mesna acts by disrupting the disulphide bonds of the mucus polypeptide chains, and is necessary in order to obtain a good quality supernatant. The amount of secretion sampled was measured by weighing the collection tube and the tube with the aliquot of PBS before and after sampling.

*Nasal challenge and sampling procedure:* Nasal challenge was carried out by using nasal aerosol application with a Heyer nebulizer[7] (Heyer Company, Germany) of the control solution (sterilized PBS, pH 7.4) and grass pollen mixtures (20 000 AU/ml, HALAB Allergy Service, Belgium). The nebulizer contained a provocation extract or the PBS. The nasal mucosa was consecutively provoked six times during 10 s (three times for each nostril alternately), the study subject being in complete apnoea after a full inspiration. This precaution prevented the provocation extract from entering the bronchial tree. The temperature of the aerosol was maintained between 28 and 30°C.

First, two consecutive samples were collected in the early morning (about 08:30 h) as a control of the baseline values. Incidentally, this provided considerable data on whether an increase of mediators was induced by the irritant action of nasal aspiration. Fifteen min later, two consecutive nasal challenges with PBS and allergen (PBS again for the control group) were performed in single blind with an interval of 15 min. Nasal secretions were then collected 5 min after each challenge. The samples were cooled on ice immediately after sampling, then mixed in a Vortex mixer, and centrifuged at 1000 × **g** at 4°C for 15 min. The supernatants were stored at –20°C until used.

*Measurement of the mediators:* The mediators of histamine and tryptase were determined by following the instructions for radioimmunoassay (RIA, RIA kits from Kabi Pharmacia, Sweden). For histamine, the total coefficient of variation (% CV) was lower than 11.9% in the working range of the assay. The minimal detectable dose of the histamine assay was 2 ng/ml; this sensitivity was sufficient for our purpose as all samples had values exceeding 7.5 ng/g. For tryptase, the detection limit is 0.5 U/l defined as the concentration indicated by the counts of three standard deviations above that of a zero sample. Between-assay variability was lower than 5% within the working range for our samples. To evaluate the possible influence of Mesna, this product was added in recovery experiments and did not show any influence on the radioimmunoassay results.

The reliability of the histamine measurement was confirmed by an additional experiment. One sample with a highest concentration of histamine from each healthy volunteer and patient was selected again, and was additionally prepared by adding the same volume of a 2.5 mg/ml diamine oxidase (Sigma, USA) solution. After incubation at 37°C for 1 h, the histamine concentration was measured again by following the same protocol.

*Objective evaluation:* The number of sneezes was recorded by absolute count. Nasal airway resistance (NAR) was measured by PAR (Heyer Company, Germany) expressed in Pa/cm$^3$/s of the left and right sides. A 100% increase of NAR at one or both nasal cavities was considered as a nasal obstruction.

*Statistical evaluation:* All statistical tests were carried out two-tailed at the 1% level of significance in order to correct for multiple comparison. The calculations were performed using the SPSS/PC+ program on an IBM compatible microcomputer. The Wilcoxon rank sum test was used to compare all the parameters with baseline value after NAC.

### Results

The study was performed without any complication such as asthma or epistaxis. All test subjects could complete their tests according to the study protocol. The amount of secretion obtained from the normal volunteers varied between 50 and 540 mg (median, 285 mg), and that from the patients, between 80 and 870 mg (median, 300 mg). It was of course easier to obtain more secretion after NAC.

None of the study subjects experienced sneezing or nasal obstruction at baseline. No significant change occurred in the number of sneezes and in nasal airway resistance before and after nasal PBS challenges in the control group and in the patient group. Sneezes were counted in 13 patients (76%) (range, 4 – 20 times) within 5 min of NAC. Seventeen patients (94%) showed nasal obstruction. Only one patient failed to show any response after NAC and was disregarded for further evaluation.

There was no significant difference in the median values for histamine and tryptase between men and women, or between the first two consecutive samples. But the baseline histamine concentration was significantly higher in the patient group (median, 36 ng/g) than in the control group (median, 19 ng/g). After adding the diamine oxidase to the samples, an average decrease of 74.9% of histamine occurred compared with the same samples not pre-treated with diamine oxidase.

*The control group:*

*Histamine.* Histamine (Fig. 1) showed a median baseline value of 19 ng/g (range, 7.5–32 ng/g). There was a significant increase in median histamine concentration to 63 ng/g (range, 22–722 ng/g) after the first PBS challenge, and 65 ng/g (range, 17–1065 ng/g) after the second PBS challenge. An overlap existed between the histamine values of the two consecutive challenges and those of the baseline.

*Tryptase.* Tryptase (Fig. 2) showed a median baseline value of 0 (range, 0–11.2 μU/g). There was a slight increase to a median value of 1.6 μU/g (range, 0–35.9 μU/g) after the first PBS challenge, and 3.5 μU/g (range, 0–56.5 μU/g) after the second PBS challenge, but these changes were not statistically significant.

*The patient group:*

*Histamine.* The concentration of histamine (Fig. 3) showed a median baseline value of 36 ng/g (range, 22–69 ng/g). A significant increase also occurred up to a median value of 69 ng/g (range, 19–543 ng/g) immediately after PBS (overlap) and to 214 ng/g (range, 77–592 ng/g) after allergen challenge (no overlap).

*Tryptase.* The median concentration of tryptase (Fig. 4) at baseline was very low, i.e., 0.1 μU/g (median, 0–6.9 μU/g). There was a significant increase only at the median concentration to 28 μU/g (range, 0–253 μU/g) after NAC (overlap).

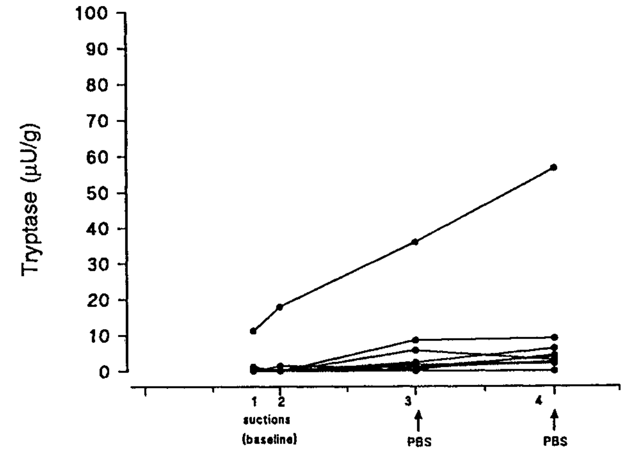

FIG. 2. Tryptase concentrations in the control group ($n$ = 10) after two consecutive PBS nasal challenges.

An additional experiment was again performed 6 months after the first experiment in five of the six volunteers of the control group in whom histamine concentration significantly increased after PBS challenge. One female subject could not participate in this confirmatory experiment due to pregnancy. In these five volunteers, four repeated nasal samples were obtained by microsuction at 15 min intervals, followed by four times PBS nasal challenge also at 15 min intervals. Nasal secretions were collected immediately after each challenge. The results showed that histamine (Fig. 5) as well as tryptase levels (Fig. 6) were not increased in the first four samples without any challenge. Histamine showed significantly increased levels after PBS nasal challenges in all the samples (no overlapping). Tryptase, however, was only slightly increased in a few samples (17.5%). In this group, no statistical evaluation was performed because of the small number of study subjects. However, there was a poor correlation between these two mediators with respect to their responsiveness to PBS nasal challenge.

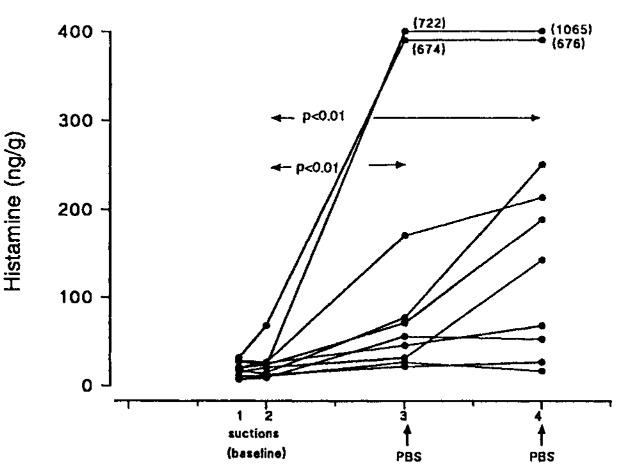

FIG. 1. Histamine concentrations in the control group ($n$ = 10) after two consecutive PBS nasal challenges.

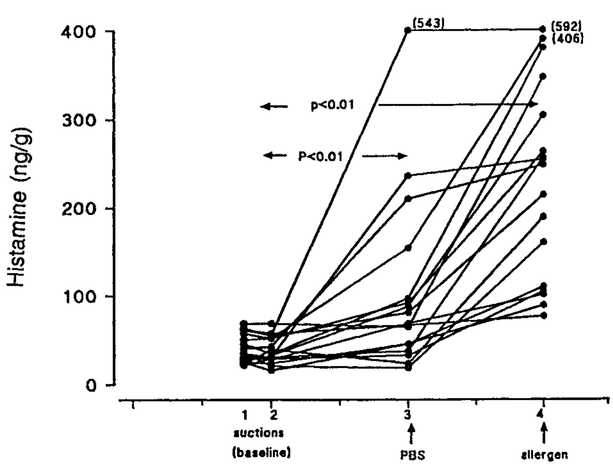

FIG. 3. Histamine concentrations in the patient group ($n$ = 17) after PBS allergen nasal challenges.

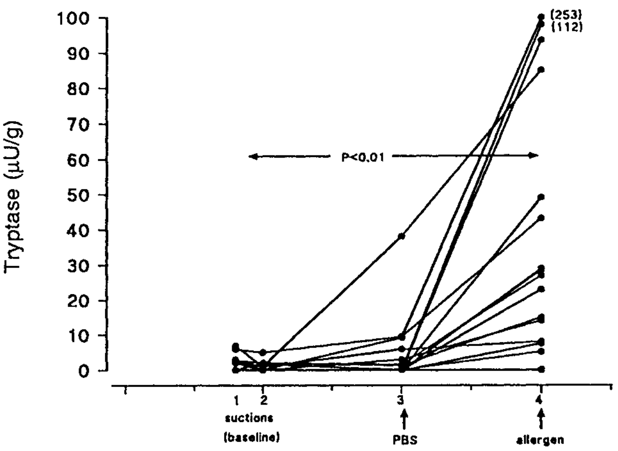

FIG. 4. Tryptase concentrations in the patient group ($n = 17$) after PBS and allergen nasal challenges.

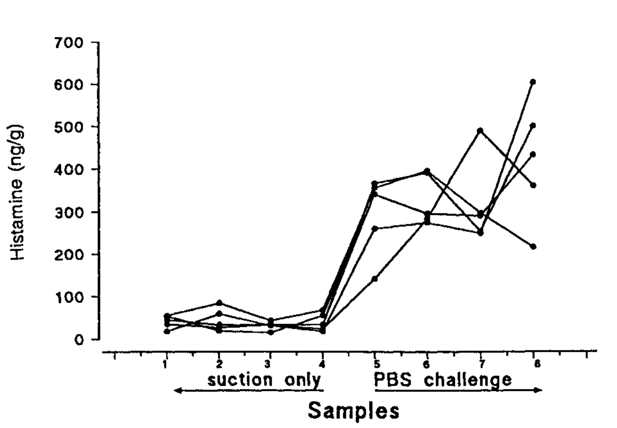

FIG. 5. Histamine concentrations in nasal secretions of five non-allergic healthy volunteers during four repeated nasal microsuctions without any challenge and followed four times by nasal PBS challenge.

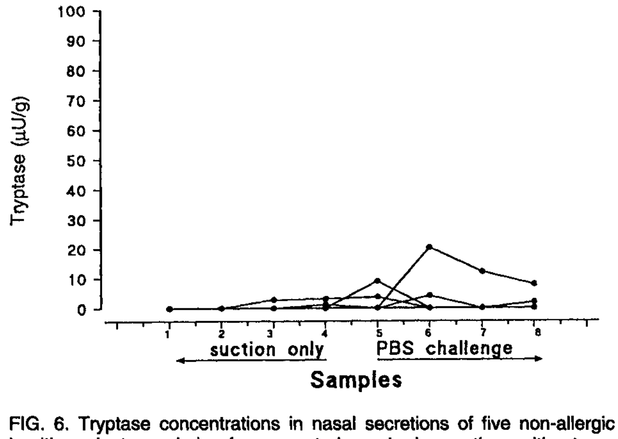

FIG. 6. Tryptase concentrations in nasal secretions of five non-allergic healthy volunteers during four repeated nasal microsuctions without any challenge and followed four times by nasal PBS challenge.

### Discussion

In recent years, it has been clearly demonstrated that mast cell activation and the release of inflammatory mediators are the initial events in the pathophysiology of the early phase in allergic rhinitis. It has also been suggested that the combined study of histamine and tryptase measurement can therefore provide useful insight into the role of mast cell activation in the pathogenesis of inflammatory responses.[3]

Until now, histamine and tryptase in nasal secretions have mostly been studied by using nasal lavage as a sampling tool. The development of nasal lavage models for the recovery of secretions on the surface of the nasal mucosa has also made it possible to identify a post-allergen challenge increase in a vast number of putative mediators of inflammation *in vivo* in humans.[8] With the lavage technique, however, it is almost impossible to determine the real concentrations of mediators in nasal secretions because the dilution factor cannot be correctly estimated. The quantitative determination of these mediators is certainly important, especially when these mediators are present in very low concentration in nasal secretions. Therefore, precise quantitative data regarding the changes in the mediator concentration can give reliable information to help understand the pathophysiology of allergic rhinitis.

By using nasal microsuction technique, the authors were able to quantify the concentration of each mediator in nasal secretions. It also allowed monitoring of the histamine and tryptase concentration in response to nasal provocation with different non-allergic stimuli such as PBS and allergen. Furthermore, it has been proved that the microsuction technique in itself does not cause any increase in mediators.

Significantly higher histamine concentrations were found in the patient group (median, 36 ng/g) than in the control group (19 ng/g). A significant increase in histamine concentration in the nasal secretions following PBS nasal challenge both in the patient and in the control group has also been demonstrated, although there existed considerable overlapping. In the patient group, after nasal allergen challenge a significant increase in histamine concentration occurred immediately without any overlapping. These increases correlate very well with nasal symptoms. The reliability of the measurement of histamine has been further confirmed by its susceptibility to diamine oxidase treatment. Our data prove that histamine can be released into the nasal secretions not only following local allergen challenge but also after PBS challenge. In the control group there was, however, poor correlation between the increase in histamine concentrations in the nasal secretions and the nasal symptoms with PBS only. In the extracellular environment and in the circulation, histamine has a half-life of less than 1 min, because of its rapid metabolism to inactive products.[9] Histamine is metabolized via two pathways, namely a diaminooxidase pathway leading to imidazole acetic acid and a methyltransferase pathway leading to

*N*-methylhistamine and *N*-methylimidazoleacetic acid.[10,11] As the sensitivity of Pharmacia histamine RIA is 18 times greater towards methylhistamine than towards histamine, it is possible that 'histamine' in this study is actually reflecting a mixture of histamine and methylhistamine.

Tryptase concentrations in almost all samples were below the minimal detection limit (0.5 μU/g). It only increased significantly in the nasal secretions of patients after nasal allergen challenge. These tryptase increases correlated very well with the concomitant histamine increases, as well as with the nasal symptoms. Furthermore tryptase was not significantly increased after nasal PBS challenge as demonstrated both in the allergic and the non-allergic group. If tryptase is only a specific marker of mast cell activation, then the increase in histamine concentrations in the nasal secretions after nasal PBS challenge cannot be explained by mast cell activation. The authors are not sure from where this histamine comes. As reported in the literature, the synthesis and storage of histamine mainly takes place in tissue mast cells and in certain white blood cells, i.e., the basophilic granulocytes. Under certain circumstances some foods contain histamine, and histamine can also be produced by bacterial flora.[12] Bacteria can trigger histamine release by immunological (IgE-dependent) and non-immunological mechanisms.[13] The mechanisms of histamine production as well as the trigger factor for the release require further study. On the other hand, our data revealed the important fact that PBS is not a reliable negative control solution, at least for nasal challenge especially when an evaluation of histamine concentration is involved.

In summary, it is obvious that the rise in histamine concentrations can be caused not only by IgE-mediated mast cell activation but also by other unknown mechanisms. Tryptase is a more reliable marker than histamine, both in the qualitative and quantitative monitoring of mast cell activation, especially during the early phase of nasal allergic reaction.

### References

1. Juliusson S, Holmberg K, Baumgarten CR, Olsson M, Enander I, Pipkorn U. Tryptase in nasal lavage fluid after local allergen challenge. *Allergy* 1991; **46**: 459–465.
2. Rasp G, Hochstrasser K. Tryptase in nasal fluid is a useful marker of allergic rhinitis. *Allergy* 1993; **48**: 72–74.
3. Proud D, Bailey GS, Naclerio RM, *et al.* Tryptase and histamine as markers to evaluate mast cell activation during the responses to nasal challenge with allergen, cold, dry air, and hyperosmolar solutions. *J Allergy Clin Immunol* 1992; **89**: 1098–1110.
4. Beaven MA, WoldeMussie E. Histamine in body fluids: its measurement in different clinical states. In: Settipane GA, ed. *H1 and H2 Histamine Receptors*. Rhode Island: OceanSide Publications, 1988; 1–32.
5. Naclerio R, Togias A, Flowers B, *et al.* Nasal lavage: a technique for elucidating the pathophysiology of allergic rhinitis. In: Mygind N, Pipkorn U, Dahl R, eds. *Rhinitis and Asthma*. Copenhagen: Munksgaard, 1990; 213–221.
6. Beiwenga J, Stoop AE, Baker HE, *et al.* Nasal secretions from patients with polyps and healthy individuals, collected with a new aspiration system: evaluation of total protein and immunoglobulin concentrations. *Ann Clin Biochem* 1991; **28**: 260–266.
7. Clement PAR, Van Dishoeck A, Van de Wal J, Stoop P, Hoek T, Van Strick R. Nasal provocation and passive anterior rhinomanometry (PAR). *Clinical Allergy* 1981; **11**: 293–301.
8. Pipkorn U. Mediators and nasal allergy. *Clin Exp Allergy* 1989; **19**: 585–589.
9. Church MK, Caulfield JP. Mast cell and basophil functions. In: Holgate T, Church MK, eds. *Allergy*. London: Gower Medical Publishing, 1993; 5.1–5.12.
10. Douglas WW. Autacoids. In: Gilman AG *et al.*, eds. *The Pharmacological Basis of Therapeutics*. New York: MacMillan, 1984; 608–646.
11. Schayer RW, Cooper AD. Metabolism of $C^{14}$ histamine in man. *J Appl Physiol* 1980; **9**: 481–483.
12. Keyzer JJ. Determinations of histamine and some of its metabolites and their clinical applications. *Pharm Weekly Sci* 1984; **6**: 218–220.
13. Norn S, Jensen C, Jarlov JO, Stahl Skov P, Espersen F, Permin H. Mediator release due to microorganisms. In: Mygind N, Pipkorn Ulf, eds. *Allergic and Vasomotor Rhinitis: Pathophysiological Aspects*. Copenhagen: Munksgaard, 1987; 140–148.

ACKNOWLEDGEMENTS. This study is supported by grants from the Free University Brussels (OZR VUB). Project number 1923320280. The authors wish to thank Michel Callewaert and Johan Schiettecatte from the RIA laboratory for their excellent technical help as well as Mr P. J. Lottefier and P. Janssens for their contributions to this article.

**Received 28 September 1994; accepted in revised form 18 November 1994**
